# COVID-19 Pandemic Experiences and Maternal Stress in Neonatal Intensive Care Units

**DOI:** 10.3390/children9020251

**Published:** 2022-02-13

**Authors:** Carmina Erdei, Natalie Feldman, Amanda Koire, Leena Mittal, Cindy Hsin Ju Liu

**Affiliations:** 1 Department of Newborn Medicine, Brigham and Women’s Hospital, Boston, MA 02115, USA; chliu@bwh.harvard.edu; 2 Harvard Medical School, Boston, MA 02115, USA; nsfeldman@bwh.harvard.edu (N.F.); akoire@bwh.harvard.edu (A.K.); lmittal@bwh.harvard.edu (L.M.); 3 Department of Psychiatry, Brigham and Women’s Hospital, Boston, MA 02215, USA

**Keywords:** parent mental health, COVID-19 grief, anxiety, NICU

## Abstract

COVID-19 compounds the already high levels of psychological distress experienced by NICU mothers. We aimed to describe the rates of NICU-related maternal stress during the COVID-19 pandemic and to determine how COVID-19 experiences correlate with high levels of stress experienced by NICU mothers. We conducted a cross-sectional analysis based on responses to a nationwide online survey to understand the relationship between COVID-19-related experiences and the stress experienced by mothers of infants admitted to U.S. NICUs (*n* = 108) during the pandemic. Results indicate that 61.9% of surveyed mothers reported experiencing high levels of stress on the Parental Stressor Scale: NICU. COVID-19-related grief was significantly associated with higher levels of maternal stress, as it related to seeing the baby’s appearance and behavior in the NICU and exposure to sights and sounds within the NICU environment. No significant associations were noted between parental stress and COVID-19-related health worries or worries about resources. Of note, our recruitment relied on convenience sampling, limiting the generalizability of study results. In conclusion, mothers who experience COVID-19-related grief appear to be more vulnerable to NICU-related stress. Prioritizing parent involvement and enhancing psychosocial support are essential strategies to mitigate the long-term consequences of heightened stress for NICU families.

## 1. Introduction

The coronavirus disease 2019 (COVID-19) pandemic has been a major disruption for individuals across the world, with elevated rates of mental health symptoms observed since March of 2020, when the U.S. outbreak first occurred [1,2]. These increased mental health concerns have been observed in the general population [3] as well as in high-risk populations, such as perinatal women [4,5].

Parents of infants hospitalized in the Neonatal Intensive Care Unit (NICU) experience increased rates of emotional distress and mental health problems in the perinatal period [6]. This is driven by multiple factors, including parent separation from the infant, who requires intensive care in an unfamiliar, highly medicalized environment; alteration of parental role; concern about a threat to the infant’s life; postpartum status of the parents; and competing responsibilities that NICU families are likely to experience. These stressors can accumulate and result in clinically significant mental health outcomes for parents. In one study, over half of mothers of NICU-hospitalized infants experienced increased stress, and over a third endorsed depressive symptoms in the clinically-significant range [7]. Another study indicated that 35% of mothers with neonates admitted to the NICU experienced acute stress disorder, which progressed to post-traumatic stress disorder (PTSD) in 8% of the cohort [8].

Though both parents are affected by their child’s admission to the NICU, mothers have been shown to express higher levels of stress [9,10], anxiety [11], and higher risk for PTSD [8,10] during and after the NICU experience as compared with fathers. Specifically, alteration of parental role has been noted to be a major driver of NICU-related stress for NICU mothers [7,9]. The perinatal period has been identified as a time of higher risk for emotional complications for women even before taking into account the possibility of a NICU hospitalization for the infant [12,13]. Moreover, a substantial number of women with pregnancy complications and subjectively traumatic births, both of which are associated with NICU hospitalization, are at risk for developing mental health difficulties [14,15]. As such, enhanced psychological support for parents of hospitalized infants has been proposed [16,17] given the potential negative consequences of altered perinatal mental health not only for the mother’s health [18] but also for the mother–infant bonding process [19] and child’s development and behavior [20,21].

In this context, the COVID-19 pandemic is an added stressor for new mothers [4], compounding the already high levels of psychological distress experienced by NICU families [22,23]. As well, there is concern that the NICU parent experience has been impacted by the necessary COVID-19-preventive precautions [24]. Following infection-control guidelines, many parents have faced traveling restrictions, decreased access to community supports, and restrictive NICU family presence policies. Institutions and intensive care units may have limited the presence of non-essential staff and reduced or eliminated on-site peer support groups during the pandemic [24]. Essential family-centered developmental care interventions and practices were disrupted, with active parental participation in the care of their infants consequently decreased. Moreover, in a recent report, nearly half of NICUs surveyed reported reductions in services, such as allied health and social work staff [25], who typically play an essential role in identification and initial support of parents who experience psychosocial distress.

In addition to the above challenges, the COVID-19 pandemic has produced new sources of worries that have been shown to be associated with concerning mental health outcomes [1]. For the purpose of this work, we focused specifically on COVID-19-related experiences, inclusive of health worry, worries related to access to resources, and grief associated with lost experiences during the pandemic, and their relationship to self-reported stress of parents who have an infant hospitalized in the NICU. These experiences generally refer to feelings regarding missing out on significant life events, limited support of family and friends due to social distancing, and loss of resources or community support. These types of COVID-19-related experiences are salient for NICU mothers given their important role as caregivers in ensuring the well-being and access to adequate resources for themselves, their infants, and their family. As well, the negative emotional experiences tied to such concerns may produce mental health symptoms and reduce a mother’s ability to respond sensitively to her infant’s needs given prior work indicating that postpartum psychological challenges may have an effect on maternal responses contingent upon infant’s cues [26,27]. Therefore, understanding the ways in which the COVID-19 pandemic has affected the experiences of mothers and infants requiring care in the NICU is highly warranted.

As such, the aims of this study were twofold: (1) to report the rates of self-reported stress of mothers whose infants were admitted in the NICU during the COVID-19 pandemic and (2) to explore how COVID-19-related experiences, inclusive of health worries, worries about resources, and grief associated with lost experiences might relate with the high stress experienced by NICU mothers. Understanding these experiences can inform effective strategies to better support families of hospitalized infants during this challenging time.

## 2. Materials and Methods

### 2.1. Participants

The sample of NICU mothers used for this analysis was drawn from the Perinatal Experiences and COVID-19 Effects (PEACE) Study, where online survey data were collected among postpartum women across the U.S. from 21 May 2020 and 23 June 2021. Participants were recruited from email listservs, social media, word of mouth, and Facebook groups. Women invited to take part in the survey included those eligible if they were at least 18 years of age and had given birth in the prior six months. The 30- to 40-min survey, which assessed maternal demographics, mental health history, and psychosocial experiences, was administered via REDCAP after obtaining informed consent. Participants were asked whether their baby had to stay in the NICU; those who indicated having NICU experiences were included in this analysis. To ensure data quality, attention checks and human verification steps were included throughout the survey and further inspected for potential response irregularities. Study procedures were approved through the Institutional Review Board at Mass General Brigham.

### 2.2. Measures

#### 2.2.1. Predictors

To assess COVID-19-related health worries, we used the Coronavirus Health Impact Survey (CRISIS) [28,29], which includes four items pertaining to concerns related to one’s health and the health of family and friends. Items assessed worries about contracting the virus, friends and family becoming infected, and one’s physical and mental health being influenced by COVID-19. Participants were asked to rate their worries on a scale of 1 to 5, with 1 being not at all and 5 being extremely. Cronbach’s alpha for item reliability was 0.85, indicating very good reliability. The sum of the items was used for analyses.

To evaluate COVID-19-related worries about resources, we administered a 6-item measure used in previous studies investigating mental health during the pandemic [30,31]. Examples of items include concerns about being able to obtain groceries, obtaining a COVID-19 test if one were to become sick, getting treatment for COVID-19 if contracted, keeping in touch with loved ones under social distancing guidelines, and maintaining employment and financial stability. Participants were asked to rate how worried they felt regarding each experience on a scale of 1 to 5, with 1 being not worried at all and 5 being very worried. Cronbach’s alpha for reliability of the items was 0.74, indicating good reliability. The sum score was used for analyses.

Finally, COVID-19-related grief was measured through a 6-item measure adapted from the Inventory of Complicated Grief [32] to capture grief experiences attributable to the pandemic [4]. These included feelings regarding missing out on significant life events, limited support of family and friends due to social distancing, and loss of resources. Additional items explored emotions, such as feeling stunned or dazed over what happened, feeling that life is empty, and feeling bitter over loss in daily routines and activities. The level to which participants agreed with these statements were assessed through a 1 to 5 scale, with 1 being strongly disagree and 5 being strongly agree. A sum score was used for analyses. Cronbach’s alpha for the items was 0.73, indicating good reliability.

#### 2.2.2. Outcomes

To assess parenting stress experience within the NICU, we administered the Parental Stressor Scale: Neonatal Intensive Care Unit (PSS:NICU), a validated measure with good internal consistency and construct validity [33]. The 26-item instrument has been employed by others to measure family stress in the NICU [7,33], with parents rating their sources of stress within 3 domains: parental role alteration (7 items), infant appearance and behavior (14 items), and sights and sounds (5 items). Participants were asked to rate their responses on a 5-point scale (“not at all stressful” to “extremely stressful”). Cronbach’s reliability for each respective subscale was 0.81, 0.86, and 0.80. The mean score for each subscale was used for analyses.

#### 2.2.3. Covariates

The covariates considered for analyses included maternal age, gestational age (GA, i.e., the difference between child’s date of birth and last menstrual period) at birth, whether the baby was current in the NICU at the survey completion (yes/no), and the length of NICU stay (i.e., the difference between infant age at hospital discharge and GA at birth). Given the increased risk for NICU stress among those with a pre-existing mental health condition, participants were asked about whether they had received a diagnosis of depression or generalized anxiety disorder (GAD) predating pregnancy. The participants could select from four options: “Yes, diagnosed and treated,” “Yes, diagnosed but not treated,” “Suspected, but not diagnosed,” and “No.” Those who reported “Yes, diagnosed” were categorized as having a pre-existing mental health disorder, and those who suspected or reported no to having a pre-existing mental health condition were categorized as not having a pre-existing mental health disorder. Given potential differences in the stress experience as a function of time over the pandemic, pandemic duration (i.e., days between the date of the declaration of COVID-19 as a pandemic in the United States on 13 March 2020 and when the participant completed the survey) was also taken into account.

### 2.3. Data Analytic Plan

We examined all covariates listed above in relation to the three subscales of the PSS:NICU (parental role alteration, infant appearance and behavior, and sights and sounds). Maternal age, length of NICU stay, whether the mother was breastfeeding at the time of survey, pre-existing depression, and duration of the pandemic were not observed to be significantly related with these outcomes. We therefore retained GA, pre-existing GAD, and whether the infant was admitted in the NICU at the time of the survey as covariates in the hierarchical multiple regression models. Each of the three PSS:NICU outcomes were regressed on these covariates (Block 1) and COVID-19-related health worries, COVID-19-related resource worries, and COVID-19-related grief (Block 2). Analyses were performed using SPSS 27.0 statistical software.

## 3. Results

Please refer to Table 1 for a summary of maternal characteristics for our sample and to Table 2 for clinical characteristics of their infants. The average maternal age was 33.5 years, and 92.6% of participants were white. Among our sample, 27.8% of women reported a pre-existing diagnosis of depression, and 28.7% reported a pre-existing diagnosis of GAD. The average GA of infants at discharge from the NICU was 37 weeks. Infants had an average length of NICU stay of 2.2 weeks (or 15.4 days), and 43.5% of them were born before 37 weeks of gestation. Of note, only one mother in our sample reporting having COVID-19 in the previous 6 months, and no infants were hospitalized in the NICU specifically due to COVID-19.

**Figure 1:**
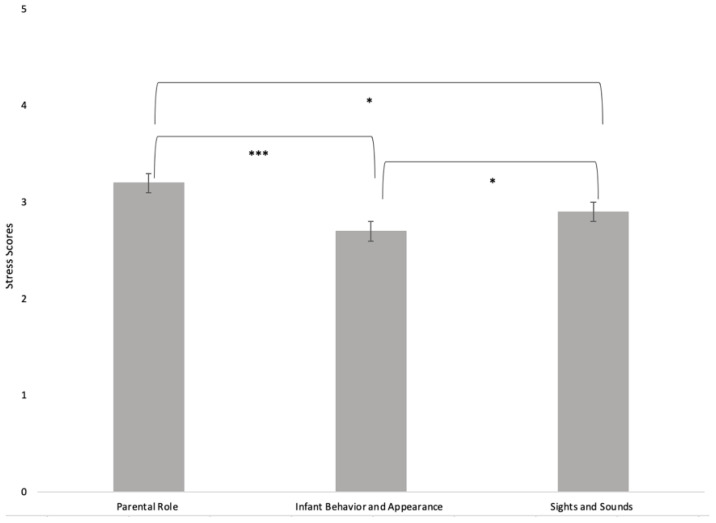
Maternal self-reported stress mean scores on the PSS:NICU, presented by subscale. The parental role alteration subscale received the highest ratings. * *p* < 0.05, *** *p* < 0.001.

Table 3 presents the means and standard deviations ranges for COVID-19-related experiences as well as the total and subscale NICU parent stress scores based on maternal self-report. With respect to COVID-19-related experiences, mothers reported that they somewhat agree/somewhat disagree to health worries and grief. In contrast, there was less concern about access to resources, with mothers on average reporting being a little worried about resources. Overall, 61.9% of mothers reported experiencing high levels of stress (PSS:NICU total score of 3 or above), with the highest score noted on the parental role alteration subscale. A visual representation of the PSS:NICU mean scores by subscale is presented in Figure 1.

Table 4 displays the associations between the predictors and self-reported NICU parental stress by subscales. As outlined, COVID-19-related grief was significantly associated with self-reported parent stress, as it related to both infant appearance and behavior and also sights and sounds in the NICU after controlling for covariates, including infant GA, pre-existing maternal GAD, and infant admission in the NICU at the time of the survey. Of note, when other covariates, such as maternal age, number of days since the pandemic onset, and prior depression, were considered, they were not significant and were therefore dropped from models to conserve statistical power.

## 4. Discussion

To our knowledge, this study is the first to assess the relationship between COVID-19-related experiences and the self-reported stress of mothers of NICU-admitted infants in a U.S.-based national sample.

Our findings indicate that the majority of new mothers (61.9%) continue to experience high levels of psychosocial stress (PSS:NICU Mean Total Score at or above 3) related to their infant’s NICU hospitalization during the pandemic. This rate is consistent with the findings of another recent study reporting an increase in maternal stress rates during COVID-19 from 44% to 57% in a NICU in Switzerland [34]. In keeping with previous reports [7,9], mothers in our study reported the highest subscale scores in the parental role alteration domain. Interestingly, the sights and sounds subscale scores were higher in our cohort as compared with pre-pandemic reports [7,9,10,35], with a similar observation documented during the COVID-19 period by the Switzerland study [34].

Further, we sought to understand how COVID-19-related experiences, inclusive of health worries, worries about resources, and grief associated with lost experiences, relate with the stress experienced by NICU mothers during this period. Our data indicate that grief of lost experiences during the COVID-19 pandemic but not worries about physical health or resources correlated with greater stress levels in NICU mothers. The lack of significant concern regarding COVID-19 health worries or resources as sources of NICU stress might be, at least in part, explained by the socio-demographic characteristics of our sample (predominantly white mothers with higher household income). However, grief associated with lost experiences during the pandemic emerged as a significant source of stress, as it related to a mother seeing her baby’s appearance and behavior in the NICU and exposure to the sights and sounds of the NICU environment. It is possible that the highly technical, medicalized environment of the NICU combined with the witnessing of enhanced, necessary infection-control procedures during the pandemic may represent increased sources of stress for NICU parents who are already experiencing grief due to the pandemic. In other words, mothers experiencing COVID-19-related grief may be more sensitive to stimuli in the NICU and may benefit from skills to enhance coping with the NICU environment, such as through exposure therapy. Such interventions, along with programs focused on guided integration of families within the NICU, parent coaching on infant behaviors and contingent responses, as well as support with development of coping strategies and resiliency while in the NICU could be considered for the benefit of the family unit.

Interestingly, while our study is consistent with prior work suggesting that parental role alteration represents the highest subscale mean score on the PSS:NICU [7], we did not find that COVID-19-related experiences predicted maternal stress as ascertained by the parental role alteration domain. Thus, it is possible that factors involved in the NICU family experience that are unrelated to the pandemic continue to interfere with mothers feeling empowered in their roles within the NICU. An important strategy that has been proposed for supporting families growing into their role as essential partners in their infant’s care is prioritizing family-integrated care practices, with NICU professionals learning to coach parents in leading the care of their infant in the NICU [36]. As such, parent presence and active involvement remain essential and should continue to be prioritized in the NICU as infection-control protocols are maintained during the pandemic.

Our findings have important clinical implications. As the COVID-19 pandemic is a major stressor that amplifies the already high levels of emotional distress experienced by NICU families [22,23], enhanced psychosocial support and access to behavioral health services are necessary to mitigate potential long-term consequences for the family [18,19,20,21]. In this context, early identification of emotional distress and adequate access to behavioral health care when indicated have been recently called for [16,17]. Enhanced access to specialized perinatal mental health care becomes even more critical during the COVID-19 pandemic, when perinatal women are likely to experience worsening depressive and anxiety symptoms, exacerbated by potential difficulties accessing necessary mental health services [37].

Furthermore, prior research indicates that for both mothers and fathers, anxiety was a better predictor of parental behavior when compared with infant medical risk in the NICU, with potential to affect the parent–infant relationship [11]. In our data, maternal pre-pregnancy GAD also appears to increase the risk of a parent experiencing NICU-related stress as driven by infant’s appearance and behavior although it is important to note that this relationship did not reach statistical significance. This underscores the importance of early identification of parents at higher risk for emotional distress, inclusive of families with preexisting risk factors, and implementation of accessible treatment options. This goal could be achieved through parent mental health programs directly embedded in the NICU environment or in close collaboration with a perinatal behavioral health provider providing systematic screening for emotional concerns, along with psychotherapy and psychopharmacologic evaluation and treatment as indicated [17]. It is recommended that behavioral health treatment be integrated within the family-centered developmental care framework in the NICU through interdisciplinary partnerships between pediatric, obstetric, and perinatal mental health providers [38]. In addition to direct psychotherapy and group support for parents and caregivers, other NICU-based interventions may be considered, such as developmental, therapist-guided parent–infant interaction [9]; parent relaxation therapy [39]; or creative music therapy [40], and could also be adapted to a virtual setting where appropriate.

### Limitations

We recognize the following study limitations, which should be considered when interpreting these results. First, we acknowledge that there may be unique experiences tied to NICU hospitalizations that were unable to be captured in this study and that may be related to maternal reported COVID-19-related experiences; infant factors including medical courses as documented in hospital records; or degree of parental involvement in their infants’ care in the NICU during the pandemic. Second, our data consist exclusively of maternal self-report such that mental health symptoms and diagnoses were not verified by a clinical provider. Standardized measures were used, which provide confidence in the assessment of our constructs, and the parent report of having a baby in the NICU is more likely to be reliable than not. Of note, our approach of categorizing women based on prior established mental health diagnoses may have excluded some mothers with suspected/undiagnosed but clinically important symptoms. Third, we relied on convenience sampling; hence, our study sample was composed primarily of women who are white, educated, and with high household income. Given the disproportionate mental health burden placed on women with social disadvantages during the pandemic, it is possible our findings do not reflect the wider range of experiences of women with increased family-social risk. Therefore, caution should be taken in generalizing findings to all mothers of high-risk infants in the NICU. Finally, our data are correlational and were collected at one time point. As such, we cannot infer causality based on the variable relations obtained.

## 5. Conclusions

Overall, our data suggest that further attention and enhanced support is warranted for NICU mothers with high levels of COVID-19-related grief during the pandemic given its association with increased stress and potential impact on family outcomes. Thus, we advocate for identifying and addressing NICU-related parent stress early through screening for mental health concerns and prioritization of family-integrated, interdisciplinary strategies. Enhanced psychosocial support and increased access to behavioral health services will not only be beneficial for high-risk infants and their parents during the stressful hospitalization but might also help promote long-term health and family wellbeing beyond NICU discharge.

## Figures and Tables

**Table 1:** Demographic characteristics from Wave I of the PEACE Study, data collected between 21 May 2020 to 23 June 2021; n = 90–108.

Predictors	Means (SD, Range) or %
Maternal age (years)	33.5 (4.1, 20.0–50.0)
Maternal race	
White	92.6%
Hispanic or Latino	1.9%
Asian and Pacific Islander	2.8%
Other	2.8%
Household income (USD/year)	
<74,999	13.9%
75,000–149,999	42.6%
150,000–224,999	20.4%
>225,000	13.9%
Missing	9.3%
Currently breastfeeding	84.2%
Maternal pre-existing mental health	
Depression diagnosis	27.8%
Generalized anxiety diagnosis (GAD)	28.7%
Pandemic duration (days)	125.0 (69.0–254.0)

**Table 2:** Infant characteristics from Wave I of the PEACE Study, data collected between 21 May 2020 to 23 June 202; n = 77–108.

Key Variables	Means (SD, Range)
Gestational age at birth (weeks)	37.0 (3.3, 26–41.0)
Length of stay in NICU (weeks)	2.2 (2.6, 0–13.0)
Infant in the NICU at the time of survey	5.6%
Reason for NICU admission	
Prematurity	43.5%
Encephalopathy/need for cooling	1.9%
Infection	5.6%
Other (most common: respiratory problems)	59.3%

**Table 3:** Means and standard deviations ranges for maternal COVID-19-related psychosocial experiences and self-reported stress scores on PSS:NICU (total and by subscale) from Wave I of the PEACE Study. Data collected between 12 May 2020 to 23 June 2021; n = 97–107.

Key Variables	Means (SD, Range)
COVID-19-related experiences	
Health worries	12.9 (3.6, 4.0–20.0)
Worries about resources	13.6 (4.7, 6.0–25.0)
Grief	19.2 (3.7, 6.0–27.0)
PSS: NICU Total	3.0 (0.7, 1.1–4.6)
Parental role alteration	3.2 (0.6, 1.2–3.9)
Infant appearance and behavior	2.7 (0.9, 1.1–5.0)
Sights and sounds	3.0 (1.0, 1.0–5.0)

**Table 4:** Multiple regression predicting parent stress in the NICU in three domains based on COVID-19-related experiences; n = 95–99, † *p* < 0.1, * *p* < 0.05.

	Parental Role Alteration			Infant Appearance and Behavior			Sights and Sounds		
Blocks of Variables	β	R2	ΔR2	β	R2	ΔR2	β	R2	ΔR2
1. Covariates		0.062	0.062		0.104	0.104 *		0.042	0.042
Gestational age	−0.165			−0.204 †			−0.015		
Currently in NICU (ref = no)	−0.228 †			−0.077			−0.071		
Maternal pre-pregnancy GAD	0.095			0.197 †			0.179		
2. COVID-19-related experiences		0.101	0.040		0.178	0.075 †		0.125	0.083 *
Health worries	0.042			0.095			0.129		
Resource worries	0.048			−0.064			−0.210 †		
Grief	0.156			0.254 *			0.260 *		

## Data Availability

Not applicable.

## Abbreviations

COVID-19	Coronavirus disease 2019
GA	Gestational age
GAD	Generalized Anxiety Disorder
NICU	Neonatal Intensive Care Unit
PSS:NICU	Parental Stressor Scale NICU
PTSD	Post-traumatic Stress Disorder
